# Association of Obesity with Proteasomal Gene Polymorphisms in Children

**DOI:** 10.1155/2013/638154

**Published:** 2013-12-21

**Authors:** Sarmite Kupca, Tatjana Sjakste, Natalija Paramonova, Olga Sugoka, Irena Rinkuza, Ilva Trapina, Ilva Daugule, Alfred J. Sipols, Ingrida Rumba-Rozenfelde

**Affiliations:** ^1^Faculty of Medicine, University of Latvia, Sarlotes Street 1a, Riga 1001, Latvia; ^2^Institute of Biology, University of Latvia, Miera Street 3, Salaspils 2169, Latvia; ^3^Institute of Experimental and Clinical Medicine, University of Latvia, No. 4 Ojara Vaciesa Street, Riga 1004, Latvia

## Abstract

The aim of this study was to ascertain possible associations between childhood obesity, its anthropometric and clinical parameters, and three loci of proteasomal genes rs2277460 (*PSMA6* c.-110C>A), rs1048990 (*PSMA6* c.-8C>G), and rs2348071 (*PSMA3* c. 543+138G>A) implicated in obesity-related diseases. Obese subjects included 94 otherwise healthy children in Latvia. Loci were genotyped and then analyzed using polymerase chain reactions, with results compared to those of 191 nonobese controls. *PSMA3* SNP frequency differences between obese children and controls, while not reaching significance, suggested a trend. These differences, however, proved highly significant (*P* < 0.002) in the subset of children reporting a family history of obesity. Among obese children denying such history, *PSMA6* c.-8C>G SNP differences, while being nonsignificant, likewise suggested a trend in comparison to the nonobese controls. No *PSMA6* c.-110C>A SNP differences were detected in the obese group or its subsets. Finally, *PSMA3* SNP differences were significantly associated (*P* < 0.05) with circulating low-density lipoprotein cholesterol (LDL) levels. Our results clearly implicate the *PSMA3* gene locus as an obesity risk factor in those Latvian children with a family history of obesity. While being speculative, the clinical results are suggestive of altered circulatory LDL levels playing a possible role in the etiology of obesity in the young.

## 1. Introduction

The prevalence of excessive weight in children has dramatically increased in the past decades, with 43 million children worldwide under the age of 5 estimated to be overweight or obese [1]. Although approximately 20% of children and adolescents in the WHO European Region are overweight [2], 31.4% of all first graders in Riga, Latvia, were recently reported to be overweight or obese [3]. The trend in Latvia, therefore, certainly points to an increasing frequency of obesity in the future, consistent with predicted global tendencies.

Obesity can be broadly classified into three genetic categories [4]. Monogenic obesity is associated with a single gene mutation, insertion or deletion (e.g., a mutation in the leptin/melanocortin pathway, resulting in severe obesity). Syndromic obesity involves concurrent mental retardation, dysmorphism, and/or organ-specific developmental abnormalities, for example, Prader-Willi syndrome. In contrast, polygenic obesity results from a complex interaction of multiple genetic, social, environmental, and behavioral factors [5, 6].

Regardless of the etiology, obesity, specifically visceral fat accumulation, has been clearly shown to be a significant risk factor for adult-onset type 2 diabetes mellitus (T2DM) [7, 8]. T2DM in the young has been studied since the 1970s, prototypically in the Pima Indians of the Southwest USA, but over the last two decades, this disease has emerged throughout the developed world as a pediatric entity of great concern in its own right [9]. The majority of these patients present with obesity and acanthosis nigricans in prepubertal and pubertal ages, along with a strong family history of T2DM [7]. Conversely, obesity by itself has become a more current problem of pediatrics and among the major risk factors for development of type 2 diabetes mellitus in the young are obesity and a strong family history of T2DM.

Recent studies have implicated proteasomal gene *PSMA6* polymorphism on chromosome 14 with an elevated risk of T2DM [10]. Deregulation of the ubiquitin-proteasome pathway, which is utilized in the functional restructuring of proteins involved in the homeostasis and regulation of significant cellular processes, has been shown to contribute to the pathogenesis of several metabolic, autoimmune, neurodegenerative, and genetic diseases [11, 12]. Moreover, ubiquitinylation regulates transcription factors and nuclear receptors that mediate insulin-induced gene expression. These studies are consistent with data indicating that the ubiquitin-proteasome system is also involved in the internalization of insulin receptor substrates 1 and 2 and in insulin degradation [13]. In addition, it has been revealed that obesity may be induced by proteasomal dysfunction and endoplasmic reticulum stress-induced insulin resistance in the liver cell [14].

Consistent with these findings on a cellular level, a number of recent studies have implicated *PSMA6* polymorphisms are associated with the development of coronary artery disease [15, 16], myocardial infarction, and ischemic stroke [17], as well as a cluster of clinical disorders (e.g., hypertension, central obesity, dyslipidemia, and insulin resistance) in patients with T2DM [18].

Since polygenic obesity is a major risk factor for T2DM and given the association between *PSMA6 *and T2DM, we hypothesize an association between childhood obesity and the *PSMA6* gene as well as the *PSMA3* proteasomal gene, which interacts with the *PSMA6* gene in forming structurally linked proteasomes [19]. The aim of our study is to detect polymorphisms in *PSMA6* (14q13.2) and *PSMA3* (14q23) genes and to investigate their possible association with childhood obesity in Latvia.

## 2. Materials and Methods

### 2.1. Subjects

The subjects in this case-control study consisted of obese children who consulted an endocrinologist at the “Gailezers” Clinic of Children's Clinical University Hospital in Riga, Latvia, with the child's parent's or primary care provider's (family physician or pediatrician) sole complaint being the child's excess body weight. After referral to a secondary care specialist (endocrinologist) to exclude a comorbid disorder—including diabetes, hypertension, obstructive sleep apnea, or psychiatric disease—or chronic use of medications that could be implicated in the etiology of obesity, the child was recruited to participate in the study before therapeutic intervention was begun. A diagnosis of obesity was established in accordance with WHO criteria, where the body mass index (BMI in kg/m^2^), calculated and applied to age- and height-specific and gender-appropriate charts, was at least at the 97th percentile [20]. Therefore, the inclusion criterion in the study was BMI defined as obese according to the WHO, with exclusion criterion being the presence of any chronic disease or condition that could elevate BMI or active attempts or therapeutic measures to treat the obesity beforehand.

The study consisted of initial informed consent, followed by a questionnaire-based interview concerning family health history, a subsequent physical examination, and blood draw. Data from the subjects was collected from 2007 through 2009 (*n* = 94, 1–17 years of age, 56% boys, 44% girls).

Prior to the study, written informed consent was given by a parent or the child's legal guardian, if the child was under 14 years of age; adolescents 14 years and older signed the consent form by themselves in addition to their parent or legal guardian. Oral responses to the questionnaire were provided by the parents or legal guardian if child was under the age of 14, or by the adolescent if the subject was 14 years or older. The questionnaire, administered by a physician, included items about the subject's lifestyle; metabolic and endocrine diseases, rheumatic, oncologic, cardiovascular, and allergic disorders; and the regular use of medications. Included were specific sections on the family's history of obesity, incorporating questions on whether siblings, parents, uncles, aunts, or grandparents are/were overweight or obese, according to the respondent.

Physical examination included measurement of body height, weight, and waist and hip circumference, followed by fasting blood samples obtained in certified procedure rooms by certified pediatric nurses before any intervention to treat the obesity was initiated.

The control group for genetic analysis was a standard reference population of Latvian inhabitants consisting of blood samples from 191 non-obese adult patients (59.5% males, 40.5% females) who were hospitalized in various wards of “Bikernieki” Clinic at Riga Eastern Clinical University Hospital and who denied any history of childhood obesity, autoimmune disease, cardiovascular disorders, or T2DM. Preliminary anthropometric and clinical data (mean ± standard deviation: age = 54.8 ± 18.6 years, BMI = 25.7 ± 3.8 kg/m^2^, glucose = 5.3 ± 0.9 mmol/L, total cholesterol = 4.3 ± 1.0 mmol/L, bilirubin = 12.6 ± 7.4 micromol/L, creatinine = 92 ± 30 micromol/L, BP_sys_ = 129.0 ± 12.8 mmHg, BP_dia_ = 80.2 ± 7.1 mmHg) supported their suitability for this genetic study. The use of non-obese adult controls who were not overweight or obese in childhood is appropriate, as it precludes incorrect classification of age-matched control group members who might develop obesity in adolescence or adulthood. Moreover, lean adult populations as controls for childhood genetic obesity studies have been successfully used previously [21], as have older underweight populations [22] that reported low weight during adolescence.

The study protocol was approved by the Central Medical Ethics Committee of the Republic of Latvia Ministry of Health.

### 2.2. Measurements and Anthropometric Parameters

All instruments were certified and calibrated before the study began.

Body height was measured without shoes, using a wall-mounted stadiometer to the nearest 0.1 cm. Body weight was measured without shoes, in underwear, using a beam scale with maximum weight of 140 kg to the nearest 0.1 kg. BMI was calculated from these data, as was each child's excess weight (by comparison with the age-, height- and gender-specific median weight given in normalized tables).

Waist circumference was measured at the end of normal expiration to the nearest 0.1 cm with inelastic measuring tape midway between the lowest rib and the superior border of the iliac crest. Hip circumference was measured with the subject standing with feet together (as close as possible) and relaxed, with the measuring tape positioned horizontally to the nearest 0.1 cm.

### 2.3. Clinical Tests

Blood (2–6 mL) was obtained from a peripheral vein and was analyzed by the clinical laboratory of the Children's Clinical University Hospital in Riga throughout the study. All biochemical parameters were determined in serum. Glucose concentration was measured using the hexokinase method. Insulin and C-peptide were determined using immunochemiluminometric assays.

Circulating triglyceride (TG), total cholesterol (TC), high-density lipoprotein cholesterol (HDL), and low-density lipoprotein cholesterol (LDL) levels were determined using the enzymatic method.

### 2.4. DNA Extraction and Genotyping

DNA was extracted from peripheral blood using a kit for genomic DNA extraction from nucleated blood cells (Fermentas, Vilnius, Lithuania). DNA was tested by electrophoresis.

After DNA extraction all samples underwent polymerase chain reaction (PCR) analysis. Polymerase chain reaction was performed with total volume 30 *μ*L, containing 100 ng genomic DNA, 10 mM dNTP Mix (Fermentas), 10 pM Reverse and forward primers, 10x Dream Tag polymerase buffer (Fermentas), 0.75 units of Dream Taq polymerase (Fermentas, Vilnius, Lithuania), and distilled H_2_O. PCR was denatured at 95°C for 7 minutes, then 35 cycles of 30 s at 94°C, 30 s at 50°C, and 30 s at 72°C, with a final extension of 7 min at 72°C, and then cooled to a final temperature of 4°C (Gene Amp PCR System 2700 Applied Biosystems). Afterwards the PCR product was tested by electrophoresis using 1% or 1.5% agarose gel.

Allele-specific PCR was performed using allele-specific forward primers differing only in the 3′-end polymorphic nucleotide and the reversal primer was common. Restriction was then done at 37°C for 24 h in a total volume of 25 *μ*L using DNA, ×10 buffer Tango with BSA Y, Enzyme-RsaI (Fermentas), and distillated H_2_O. The restriction was tested by electrophoresis using 1% or 1.5% agarose gel.

The SNP natures of the polymorphisms in our candidate genes—*PSMA6 *gene locus rs2277460 (c.-110C>A), *PSMA6 *gene locus rs1048990 (c.-8C>G) and *PSMA3* gene locus rs2348071 (c. 543+138G>A)—are represented in Table 1, respectively.

DNA extraction and genotyping was performed according to previously published protocol [23].

### 2.5. Statistical Analysis

Descriptive statistics were calculated for each parameter and compiled for the entire case group. Body weight exceeding expected norms was calculated for each child individually and averaged in all 94 children.

Data analysis was made using the PAST (Palaeontological Statistics, version 1.63) program. The significance of differences between groups was determined using the *χ* ^2^ test (*P* < 0.05). To estimate the significance between genotype groups, a *t*-test and one way ANOVA were used. Results were considered statistically significant if the *P* value was less than 0.05 (*P* < 0.05).

## 3. Results

The subjects included 94 children with pronounced obesity. To characterize the entire group, we determined certain anthropometric and clinical values (Table 2). With the exception of the anthropometric parameters, the clinical values of the subjects did not reveal any underlying pathology concurrent with the obesity and were within the normal range for such indicators.

Table 3 summarizes all genotyped results. We analyzed two SNPs of the *PSMA6* gene and one of the *PSMA3* gene in our subjects by comparing the polymorphism frequency with that of the most common allele homozygote—defined as the most common allele in the reference Latvian population to be homozygous. *PSMA3*, *PSMA6 *c.-110C>A, and *PSMA6* c.-8C>G SNPs showed no significant difference in frequency between the obese children and normal-weight controls, although a trend suggesting a relationship between *PSMA3* polymorphisms and obesity was apparent.

To examine possible differences in polymorphism frequency as a function of heritability, we divided our subjects into two subgroups, one comprising subjects with a family history of obesity (*n* = 59) and other consisting of those with no reported history of family obesity (*n* = 32). Comparing these two subgroups with each other (Table 2), no differences in anthropometric or clinical measurements were observed. While it could be claimed that the subgroup denying familial history of obesity appears less obese anthropometrically, the small number of subjects and the wide range of values (as denoted by large standard deviations) precluded statistical confirmation of this impression. As with the massed clinical parameters, no significant blood pressure differences were detected at any age between the subgroups with dissimilar history of familial obesity. Comparing each of these subgroups individually to the controls with respect to gene polymorphisms (Table 4), no differences in SNP frequency were found at either the *PSMA6 *(c.-110C>A) or *PSMA6* (c.-8C>G) locus in either children's subgroup, although a trend was detected in *PSMA6* (c.-8C>G) polymorphism frequency differences in the subgroup denying familial obesity, in comparison to the controls. However, a highly significant difference was observed between the subjects affirming a family history of obesity and non-obese controls in SNP frequency at the *PSMA3* locus (*P* < 0.01). *PSMA3* polymorphisms were not significantly associated in the subjects denying a history of familial obesity.

While a significant association of SNPs with any of the other measured variables (i.e., age, height, weight, BMI, excess weight, waist circumference, hip circumference, waist-to-hip-ratio, glucose, c-peptide, insulin, cholesterol, triglycerides, LDL-cholesterol, or HDL-cholesterol) in the subjects was not observed, there was one notable exception. The mean values of low-density lipoprotein cholesterol in the obese subjects were significantly different (*P* < 0.05) among the three genotypes (GG = 2.40 ± 0.16 mmol/L, GA = 2.20 ± 0.09 mmol/L, and AA = 3.31 ± 0.45 mmol/L) at the *PSMA3* locus irrespective of family obesity history.

## 4. Discussion

We investigated the possible association of polygenic obesity with single nucleotide polymorphisms of the *PSMA6 *and *PSMA3* proteasomal genes, based on our hypothesis that altered proteasome expression may be an important factor that can result in obesity in children. Candidate genes were selected based on previous reports describing a statistically reliable association between T2DM and proteasomal genes in comparison to nondiabetic controls and the association of proteasome expression with elevated BMI and obesity. We therefore investigated SNPs in proteasomal genes to test the hypothesis that the polymorphisms of proteasomal genes could play a role in the development of polygenic obesity. The *PSMA6* gene was included in this study based on previous reports [10]. The *PSMA3* gene was also selected, based on evidence of interaction between *PSMA6* and *PSMA3* genes, according to the GeneCards database and National Center for Biotechnology Information. To our knowledge, this is the first study investigating a possible association between proteasomal genes on chromosome 14 and obesity in children. We focused our attention on three loci—*PSMA6* c.-110C>A, *PSMA6* c.-8C>G, and *PSMA3* c. 543+138G>A—to study the association between these particular genes and childhood obesity in Latvia, as well as associations between genotypes and clinical parameters other than elevated age-appropriate body weight.

The polymorphism frequencies of the *PSMA6* c.-110C>A gene in the subjects did not differ from those in the reference group, and therefore we did not confirm the relevance of *PSMA6* c.-110C>A gene SNPs and obesity in children. Similarly, we did not establish a clear association between SNPs located in *PSMA6* c.-8C>G and childhood/adolescent obesity. However, while *PSMA3* polymorphism frequency did not reach the level of significance in association with obesity of the entire case group, our results suggest a trend that certainly bears future investigation. As reported in previous studies, high proteasome levels are risk factors for the development of human obesity [24]. If an elevated BMI is observed in individuals with more frequent proteasome variations, we could justifiably assume an association between the quality of the resultant proteasome and the severity of obesity or that the functional proteasome concentration might be higher due to a change in a single nucleotide and thus correlated with degree of obesity.

Since previous studies have clearly implicated that family history plays an important role in childhood obesity [9], we wanted to examine the possible effect of familial influences on childhood obesity, dividing our case group into two subgroups, one subgroup reporting a family history of obesity and one subgroup denying a family history of obesity. Although polymorphisms in either of the *PSMA6 *gene loci were not implicated in the development of childhood obesity in the children with family history of obesity, the differing frequencies of SNPs in the *PSMA3* gene between the reference group and those with a family history of obesity indicated a very robust association. While it is premature to speculate about the relevance of the preponderance of the GA genotype (64% frequency) in those with a family history of obesity, as opposed to the reference group (35% frequency), it appears that base-pair variations at this locus are consistent with proposed genetic mechanisms involved in the etiology of obesity.

On the other hand, the limits to conferring an unambiguous role for polymorphisms in the *PSMA3* gene for the development of obesity are clearly evident from the results of the subject subgroup denying a family history of obesity. No reliable difference in SNPs at this locus was detected between this subgroup and the control group, notwithstanding the obesity of the subjects. One possible interpretation is that the two subgroups, while similarly obese in terms of identified anthropometric and clinical parameters, may have been presenting with etiologically different forms of obesity—one driven to a certain extent by heritability and the other where environmental factors predominated in promoting overweight. While the results with *PSMA3 *SNPs are consistent with such a hypothesis, the suggestive association of SNP frequency differences at the *PSMA6* c.-8C>G locus only in the subgroup *not reporting* familial obesity, in comparison to the control group, is clearly counterintuitive, unless one was to speculate about SNPs having taken place at that locus starting with the generation being studied. An SNP frequency examination of the subjects' first-degree relatives would shed some light on these discrepancies and would seem appropriate in the future in order to clarify the specific roles played by polymorphisms at the *PSMA3* and *PSMA6* c.-8C>G loci in the promotion of obesity. Additionally, it must be emphasized that inclusion of obese subjects into different subgroups according to family history of obesity was based solely on oral responses to interviewer's questions, therefore not independently verifiable and admittedly open to differences in interpretation, accuracy of memory, and other subjective factors. The lack of objective evaluation and verification of obesity in first- and second-degree relatives, or more accurately, verification of *absence* of obesity among relatives, dictates that the results considering the role of family history should be viewed with some caution.

In contrast, SNP frequency differences at the *PSMA6* c.-110C>A locus were not associated in either of the subgroups in comparison to the reference group, a result consistent with the notion that genetic influences on the development of obesity are not to be found at this particular site on chromosome 14. Nevertheless, the results from case subgroup with family obesity history strongly implicate *PSMA3* polymorphisms with childhood obesity, leaving aside for the moment the ambiguities of mechanisms and heritability that will need to be addressed in future studies.

It has long been known that elevated BMI in childhood greatly increases the risk of coronary heart disease in adulthood and is associated with an increased risk of acute coronary syndrome [25–28]. Along with previous studies that have demonstrated an association between SNPs at the *PSMA6 *rs1048990 and *PSMA6* rs12878391 loci and myocardial infarction and coronary artery disease [29], our results are consistent with the hypothesis that proteasomal gene mutations may play an important role in the development of coronary artery disease.

Studies of polymorphisms on chromosome 16 have found significant genotype associations with weight, BMI, waist circumference, and waist-to-height ratio [30]. We did not find similar associations between SNP frequency differences and weight, BMI, excess weight, waist circumference, hip circumference, waist-to-hip-ratio, glucose, c-peptide, insulin, cholesterol, triglycerides, or HDL-cholesterol among the three chromosome 14 proteasomal genes studied. However, the association between polymorphism frequency differences at the *PSMA3* locus with circulating low-density lipoprotein cholesterol levels is intriguing. Disturbances in LDL levels are one among the major hallmarks of overweight, with elevated low density lipoprotein cholesterol associated with the dyslipidemia, insulin resistance, and cardiovascular disease prevalent in visceral obesity [31]. While the case subjects in our study were nondiabetic, had lipid levels in the normal range, and did not present with signs of cardiovascular impairment (e.g., all were normotensive, not exceeding the 95th systolic or diastolic blood pressure percentile appropriate for their age, height, and gender), their history of weight dysregulation was relatively recent (years) as opposed to the decades required for the full effects of dyslipidemia to become apparent. Nevertheless, the pronounced obesity levels apparent in the adolescent subgroups in our study are a clear forerunner for dyslipidemia-related health issues that await these subjects in the future. Since, to our knowledge, there have been no previous studies of SNPs and obesity in children analyzing the association of proteasomal gene polymorphisms with lipoprotein levels, the results support further investigation focusing on the nature of the relationship between *PSMA3* polymorphisms and LDL-cholesterol levels, both developmentally and clinically.

Our results provide clear evidence of an association between *PSMA3* c. 543+138G>A single nucleotide polymorphisms and pronounced childhood obesity in Latvia that at least has a familial component. This evidence appears to characterize a specific relationship between proteasome gene structure and accumulation of fat mass, with possible etiological mechanisms still being unclear, as none of the other clinical parameters included in our study that could be co-morbid with obesity demonstrated such an association. The singe exception, however, is the association of *PSMA3* gene SNPs with low-density lipoprotein cholesterol levels in the entire case group. Although it is attractive to speculate that *PSMA3* is involved in regulation of LDL cholesterol levels and such genetically-regulated changes in LDL levels are consistent with dyslipidemia being a major cofactor in the etiology of obesity, it is still premature to propose LDL differences as the mechanism involved in development of obesity in our case group subjects. While the significance of these differences remains to be elucidated, it must be emphasized that LDL levels were within the normal range for each subject; hence, the children did not present with dyslipidemia in addition to obesity. Moreover, while the *PSMA3* association with obesity per se was not statistically established for the entire case group, a trend in that direction was apparent. This association was statistically confirmed when considering only those children affirming a family history of obesity, with the lack of such a relationship between *PSMA3* polymorphisms and obesity in the subjects denying obese first- and second-degree relatives indicating that these nucleotide changes may play a permissive or predisposing, as opposed to an obligatory, role in the etiology of obesity.

It should be noted that, while this study's hypothesis was based on previous reports in the literature investigating *PSMA6* and *PSMA3* gene SNPs in Chinese and other Far East populations, globalization trends make it more likely that clinicians in the future will serve ever more ethnically diverse patient populations. Our results using a Latvian population indicate that SNPs of the *PSMA3* gene may well be a harbinger of obesity-related disorders in, at least, Eastern European populations as well. In practical terms, the comparatively elevated circulating LDL cholesterol levels associated with *PSMA3* gene SNPs in obese children, while still in the normal range, are nevertheless a cause of concern that may well provide practicing physicians with a possible predictor of future disorders. Such “high-normal” clinical values are used extensively to foreshadow the development of subsequent disorders (e.g., hypertension) and rising LDL levels in an already obese pediatric population may indicate the presence of a genetic predisposition to lipid disorders and predict the eventual emergence of frank dyslipidemia comorbid with the obesity. Therefore, our results suggest that vigilant observation of LDL levels, while still in the normal range, may well provide the practicing physician with additional information to evaluate and therapeutically manage or decrease the severity of a notable risk factor associated with obesity.

In summary, our study demonstrates an association between single nucleotide polymorphisms at the *PSMA3* locus and obesity in children reporting a family history of obesity. Further studies are necessary to more completely understand the possible role base pair changes at this site play in the etiology of obesity and the comorbidities associated with this disease.

## 5. Conclusion

*PSMA3* rs2348071 (c. 543+138G>A) gene SNPs are associated with the genotype of obese children having a family history of obesity in comparison to a non-obese reference group of adults.

*PSMA3* rs2348071 (c. 543+138G>A) gene SNPs are associated with circulating low density lipoprotein cholesterol levels in obese children, in comparison to a non-obese reference group.

In contrast, no association between *PSMA6 *rs2277460 (c.-110C>A) gene SNPs and other measured clinical parameters (including blood pressure, glucose, insulin, C-peptide, triglyceride, total cholesterol, and high density lipoprotein cholesterol) was observed in obese children in comparison to the non-obese reference group.

However, while *PSMA6 *rs1048990 (c.-8C>G) gene SNPs were not associated with obesity in children in comparison to the non-obese reference group, a trend indicating a possible association was observed in the subgroup of obese children not reporting a family history of obesity.

## Figures and Tables

**Table 1:** General information about the genotyped loci.

Gene/chromosome location	Polymorphism			Genotyping method	Primers	Fragment size (bp)
	Variation	ID	Function			
*PSMA6*/14q13.2	c.-110C>A	Rs2277460	Promoter	ASA	F: 5′-ATGCAAGAGCGGAAGAAAC-3′	256
					F: 5′-ATGCAAGAGCGGAAGAAAA-3′	
					R: 5′-CTGAATTGCCCTGTCATGGTA-3′	
	c.-8C>G	Rs1048990	5′UTR	CAPS/*RsaI *	F: 5′-ACGTTGGATGCTCCAGATGAAAGCCTGA-3′	256/161 + 94
					R: 5′-ACGTTGGATGGCCCTATCTTCCTTAACTCTC-3′	
*PSMA3/*14q23	c. 543+138G>A	Rs2348071	Int7	CAPS/*TscAI *	F: 5′-GTCTAAGGCAGGGATGTCCA-3′	232/166 + 66
					R: 5′-ACCAGCTTTCCCATTCAGTG-3′	

SNP: single nucleotide polymorphism.

ID: indicates GenBank polymorphic loci accession number.

ASA: allele specific amplification.

CAPS: cleaved amplified polymorphic site.

Bp: base pairs.

F: forward.

R: reverse.

**Table 2:** Anthropometric and clinical parameters of the case group.

Parameter	Entire case group		With family history of obesity		Without family history of obesity	
	Mean ± SD	*n*	Mean ± SD	*n*	Mean ± SD	*n*
Age (years)	11.1 ± 3.4	94	11.1 ± 3.4	59	11.6 ± 3.2	32
Height (m)	1.53 ± 0.19	94	1.53 ± 0.19	59	1.52 ± 0.18	32
Weight (kg)	66.72 ± 23.3	94	67.8 ± 23.9	59	64.8 ± 22.2	32
Excess weight (kg)	21.7 ± 12.9	94	22.8 ± 13.2	59	19.9 ± 12.4	32
BMI (kg/m^2^)	27.6 ± 4.7	94	28.1 ± 4.7	59	27.0 ± 4.8	32
Waist circumference (cm)	84.8 ± 11.7	83	86.1 ± 11.7	53	82.6 ± 11.7	29
Hip circumference (cm)	97.6 ± 13.7	83	98.3 ± 13.4	53	96.1 ± 14.4	29
Waist-to-hip ratio	0.87 ± 0.07	83	0.88 ± 0.07	53	0.86 ± 0.06	29
Glucose (mmol/L)	4.89 ± 0.64	90	4.97 ± 0.71	56	4.73 ± 0.51	32
Insulin (*µ*U/mL)	13.1 ± 32.5	86	24.1 ± 35.5	54	21.7 ± 27.7	31
C-Peptide (ng/mL)	3.61 ± 3.59	86	3.52 ± 3.05	55	3.85 ± 4.51	30
Cholesterol (mmol/L)	4.22 ± 0.81	89	4.20 ± 0.73	57	4.27 ± 0.96	30
Triglycerides (mmol/L)	1.41 ± 0.63	86	1.44 ± 0.62	56	1.36 ± 0.66	29
HDL-cholesterol (mmol/L)	1.26 ± 0.28	87	1.25 ± 0.29	57	1.25 ± 0.27	29
LDL-cholesterol (mmol/L)	2.32 ± 0.77	87	2.29 ± 0.66	57	2.39 ± 0.98	29

**Table 3:** Genotype frequencies in the obese case group (*n* = 94) and non-obese control group (*n* = 191).

Gene	SNP	Genotyping results						
		Genotype	Subjects (*n*)		Frequency (%)		Significance values	
			Case	Control	Case	Control	*P*	CI 95%
*PSMA6 *	c.-110C>A	CC	83	166	88.3	86.9	0.74	0.43–1.85
		CA	11	25	11.7	13.1		
		AA	0	0	0	0		
	c.-8C>G	CC	78	158	83.0	82.7	0.43	0.59–1.97
		CG	14	32	14.9	16.8		
		GG	2	1	2.1	0.5		
*PSMA3 *	c. 543+138G>A	GG	35	102	37.2	53.4	0.0008	0.85–1.8
		GA	54	66	57.5	34.6		
		AA	5	23	5.3	12.0		

SNP: single nucleotide polymorphism.

*P* value based on *χ* ^2^ method and 10,000 permutations.

CI 95% indicates confidence interval.

**Table 4:** Genotype frequencies in obese children with (OB) and without (NOB) a family history of obesity.

Gene	SNP	Genotyping results												
		Genotype	Subjects (*n*)		Frequency (%)		Significance values		Subjects (*n*)		Frequency (%)		Significance values	
			OB	Control	OB	Control	*P*	CI 95%	NOB	Control	NOB	Control	*P*	CI 95%
*PSMA6 *	c.-110C>A	CC	51	166	86.4	86.9	0.93	0.41–2.26	29	166	90. 6	86.9	0.56	0.41–5.14
		CA	8	25	13.6	13.1			3	25	9.4	13.1		
		AA	0	0	0	0			0	0	0	0		
	c.-8C>G	CC	49	158	83.0	82.7	0.86	0.45–2.16	26	158	81.2	82.7	0.03	0.43–4.04
		CG	10	32	17.0	16.8			4	32	12.5	16.8		
		GG	0	1	0	0.5			2	1	6.3	0.5		
*PSMA3 *	c. 543+138G>A	GG	19	102	32.2	53.4	0.002*	0.17–0.60	16	102	50.0	53.4	0.77	0.36–1.77
		GA	38	66	64.4	34.6			13	66	40.6	34.6		
		AA	2	23	3.4	12.0			3	23	9.4	12.0		

SNP: single nucleotide polymorphism.

*P* value based on *χ* ^2^ method.

CI 95% indicates confidence interval.

OB: subjects with family history of obesity.

NOB: subjects without family history of obesity.

*Significance based on *P* < 0.05 and CI_95_ < 1.0.
